# *Burkholderia cenocepacia* Infections in Cystic Fibrosis Patients: Drug Resistance and Therapeutic Approaches

**DOI:** 10.3389/fmicb.2017.01592

**Published:** 2017-08-22

**Authors:** Viola C. Scoffone, Laurent R. Chiarelli, Gabriele Trespidi, Massimo Mentasti, Giovanna Riccardi, Silvia Buroni

**Affiliations:** ^1^Department of Biology and Biotechnology, University of Pavia Pavia, Italy; ^2^Respiratory and Vaccine Preventable Bacteria Reference Unit, Public Health England London, United Kingdom; ^3^Department of Microbiology, Royal Cornwall Hospital Truro, United Kingdom

**Keywords:** *Burkholderia cenocepacia*, resistance, epidemiology, antibiotics, new therapies

## Abstract

*Burkholderia cenocepacia* is an opportunistic pathogen particularly dangerous for cystic fibrosis (CF) patients. It can cause a severe decline in CF lung function possibly developing into a life-threatening systemic infection known as cepacia syndrome. Antibiotic resistance and presence of numerous virulence determinants in the genome make *B. cenocepacia* extremely difficult to treat. Better understanding of its resistance profiles and mechanisms is crucial to improve management of these infections. Here, we present the clinical distribution of *B. cenocepacia* described in the last 6 years and methods for identification and classification of epidemic strains. We also detail new antibiotics, clinical trials, and alternative approaches reported in the literature in the last 5 years to tackle *B. cenocepacia* resistance issue. All together these findings point out the urgent need of new and alternative therapies to improve CF patients’ life expectancy.

## Introduction

*Burkholderia cenocepacia* is a member of the *Burkholderia cepacia* complex (Bcc) (Vanlaere et al., 2008; De Smet et al., 2015; Ong et al., 2016; Weber and King, 2017), a group of 22 Gram negative related bacterial species widespread in the environment. It can infect cystic fibrosis (CF) patients causing a severe decline in lung function which can further develop into a life-threatening systemic infection known as cepacia syndrome. CF is the commonest autosomal recessive disease in Europe affecting 1 in 2500 newborns, nevertheless it is considered rare according to the European Union definition (i.e., disease affecting less than five individuals per 10,000 people) (Farrell, 2008). More than 2,000 mutations in the cystic fibrosis transmembrane conductance regulator (CFTR) gene are responsible for the disease. The CFTR encodes a chloride ion channel which is expressed on the surface of cells in many tissues, including lungs, gut, and pancreas; its malfunctioning causes the production of viscous secretions which are particularly dangerous in the lungs where opportunistic infections consequently occur (Knowles and Durie, 2002). It is noteworthy, that chronic respiratory infections and inflammation are the leading cause of death in CF (Ciofu et al., 2013). Moreover, colonization of immunocompromised individuals has also been reported (Ganesan and Sajjan, 2012).

Bcc bacteria, particularly *B. cenocepacia*, are naturally resistant to different classes of antibiotics used in clinical practice (Mahenthiralingam et al., 2005; Burns, 2007) and their pathogenicity is promoted by several virulence determinants (Loutet and Valvano, 2010; Sousa et al., 2011). These characteristics, together with the ability to adapt to environmental changes, make the treatment of *B. cenocepacia* infections particularly challenging. In fact, it has been shown that during long-term colonization, *B. cenocepacia* can undergo transcriptional reprogramming in response to host immune response, antimicrobial therapy, nutrient availability, and oxygen limitation (Mira et al., 2011). Consequently, a genomic approach as well as the construction of mutant libraries revealed crucial in identifying essential genes responsible for antimicrobial resistance, virulence, and adaptation (Wong et al., 2016; Gislason et al., 2017).

From an epidemiological perspective, a recent study by Salsgiver et al. (2016) reported that from 1995 to 2012 the prevalence of Bcc infections decreased from 3.6 to 3.0% in the United States (Salsgiver et al., 2016). This was ascribed to the combination of new antimicrobial therapies with CFTR correctors and potentiators, as well as the introduction of new guidelines detailing prevention and eradication strategies (Saiman et al., 2014).

Although *Burkholderia* species are relatively rare amongst CF patients, they still cause serious challenges. Indeed, despite therapeutic advances, respiratory failure remains the major cause of premature death and lung transplant the best option to treat the most severe cases (Ramos et al., 2017). However, a main post-transplant complication is represented by infection with multidrug resistant bacteria and Bcc was recognized as a significant contributor to CF morbidity and mortality associated to increased post-transplant death rate (Alexander et al., 2008; Chaparro and Keshavjee, 2016). In this context, studying the resistance profile, the mechanisms underlying resistance, as well as the epidemiology of CF pathogens seems essential to improve management of these infections.

In this review, we describe the clinical distribution of *B. cenocepacia* resistant strains reported in the last 6 years and the methods for identification and classification of epidemic strains. All together these data point out the urgent need of new and alternative therapies to treat Bcc infections and improve CF patients’ life quality and expectancy. Finally, we summarize new antibiotics, clinical trials, and alternative approaches reported in the literature in the last 5 years to tackle *B. cenocepacia* resistance to antimicrobials.

## Epidemiology of *B. cenocepacia* Strains

The frequency of Bcc infections in CF patients is quite variable. *B. cenocepacia* and *Burkholderia multivorans* are the most commonly isolated in Australia, New Zealand, and several European countries. *B. multivorans* is currently the most prevalent in the United States and Canada^1^^,^^2^. Moreover, *Burkholderia gladioli*, a non-Bcc member, is increasingly isolated and has become the third most common *Burkholderia* species in the United States, but it is not common in European CF patients^2^^,^^3^ .

It is worth mentioning that following to the recommendations for infection prevention and control in CF, published in 2003 and updated in 2013 (Saiman et al., 2014), strategies to reduce the risk of patient-to-patient transmission and acquisition from the environment were implemented and a progressive decrease in Bcc prevalence was subsequently observed (Salsgiver et al., 2016). Transmission of Bcc infection occurs as a consequence of both direct and indirect contact (e.g., *via* infectious droplets) between patients. Initially, social contacts during summer camps, very popular among CF patients until 1998, were the most likely cause of Bcc cross-infections. Nevertheless, a *B. cenocepacia* strain isolated from soil in 2000 was shown to be indistinguishable from clinical isolates by different typing methods, thus demonstrating that acquisition of Bcc from the environment could occur, and explaining why the incidence of Bcc infection in CF patients has not been eliminated (LiPuma et al., 2002).

Several single-center or multicenter studies have suggested that poor outcomes might be a consequence of the infecting Bcc species (Murray et al., 2008). Indeed, the worst outcome was observed with *B. cenocepacia* which leads to an excessive mortality rate among CF patients (Corey and Farewell, 1996; Alexander et al., 2008; De Soyza et al., 2010; Gilljam et al., 2017). Despite advanced lung disease, several *B. cenocepacia* infected patients are not considered for lung transplant (Ramos et al., 2016).

From the end of the 1990s, epidemiological studies demonstrated that *B. cenocepacia*, at that time known as Bcc genomovar III, was the most prevalent Bcc pathogen in CF patients. Using *recA* sequence analysis, the species was then subdivided into four phylogenetic clusters IIIA – IIID, however, almost all clinical isolates belong to the IIIA and IIIB subgroups. Among them, the ET-12 strain (ST28) and the Czech strain (ST32), which spread within CF patients in Canada and Europe, belong to the *B. cenocepacia* IIIA group, while the *B. cenocepacia* strains dominant in the United States, such as the Midwest clone and the PHDC strain, are part of the subgroup IIIB (Drevinek and Mahenthiralingam, 2010). *B. cenocepacia* IIIC group is exclusively environmental, while IIID strains have been isolated from CF patients only in Sweden, Argentina, and in Italy (Manno et al., 2004; Campana et al., 2005).

These studies suggest that chronic *B. cenocepacia* infections resulted from the colonization of few clonal bacterial strains. Nevertheless, CF patients infected with genotypically similar *Burkholderia* strains can manifest different outcomes. Indeed, rapid evolution of *Burkholderia* species was demonstrated during infections or *in vitro* under stress conditions (Drevinek et al., 2010; Sass et al., 2011), suggesting that mutations can occur and accumulate in clonal lineages as a response to suboptimal growth conditions. For instance, an epidemic *B. cenocepacia* clone prevalent in the Serbian CF population (i.e., ST856) was shown to be subjected to variations in virulence and genotype as a consequence of the lung adaptation (Malešević et al., 2017).

It is noteworthy that, thanks to the prevention and control strategies introduced in the late ‘90s, novel Bcc infections occurring in Canada, the United States and many European countries are presently caused by non-epidemic *B. cenocepacia* strains or by non-clonal *B. multivorans* strains or Bcc species other than *B. cenocepacia*. The presence of non-clonal isolates of various Bcc species in CF patients, also when strict control measures are undertaken, suggests an acquisition from environmental sources rather than cross-infections. Further studies are now necessary to analyze so far unexplored environmental niches of *Burkholderia* species and then implement appropriate prevention measures (Zlosnik et al., 2015).

### Epidemiological Methods

The analysis of Bcc epidemiology, particularly of *B. cenocepacia*, in CF patients is paramount as highlighted by the several methods used for genotyping. Indeed, transmissibility markers were identified in *B. cenocepacia* epidemic strains, such as the *cblA* pilin gene (Sun et al., 1995) and the *B. cepacia* epidemic strain marker (BCESM) belonging to a pathogenicity island (Baldwin et al., 2004), or the IS1363 insertion sequence (Liu et al., 2003). However, even if some epidemic lineages are associated to these genetic markers, others were not, thus genotyping studies are still required for a full understanding of cross-transmission and for epidemiological surveillance.

Among molecular typing methods, the most used are ribotyping (LiPuma et al., 1988; Dasen et al., 1994), pulsed-field gel electrophoresis (PFGE) (Tenover et al., 1995), random amplified polymorphic DNA (RAPD) (Mahenthiralingam et al., 1996), repetitive elements PCR (rep-PCR) (van Belkum et al., 1996) and multilocus sequence typing (MLST) (Coenye and LiPuma, 2002; Urwin and Maiden, 2003; Spilker et al., 2009). Macro-restriction of chromosomal DNA followed by PFGE was considered the gold standard in bacterial typing for a long time, and was widely applied to the molecular epidemiology of Bcc (Coenye et al., 2002). However, as PFGE is quite a laborious technique and typing results are difficult to compare between different laboratories, MLST replaced it as preferred genotyping method also for CF pathogens (Saiman et al., 2014). MLST differentiates bacterial isolates by comparing the sequence of seven housekeeping gene fragments and then characterizing strains by the resulting allelic profile. For each housekeeping gene, sequence variants are designated as distinct alleles and each allele profile defines a specific sequence type (ST). Compared to other genotyping methods, MLST offers several advantages, primarily yielding unambiguous and reproducible results that can be easily and reliably compared between different laboratories. MLST profiles of more than 2000 Bcc isolates are freely available online^4^ (Jolley and Maiden, 2010), however this technique is quite expensive and time consuming, thus limiting its application for routine use in clinical microbiology laboratories or in genotyping large collection of isolates in national surveillance programs.

For the above reasons, alternative methods were developed to allow routine or large-scale analysis, such as the SNaPBcen assay targeting only single nucleotide polymorphisms in MLST genes instead of analyzing full sequences (Eusebio et al., 2013); the PCR assays based on MLST and specific for particularly globally distributed epidemic strain (Dedeckova et al., 2013); or multilocus variable-number tandem-repeat analysis (MLVA) (Segonds et al., 2015).

Evolution and adaptation of pathogens during chronic infections are of great importance for choosing the appropriate therapeutic strategy, however, the underlying molecular bases for Bcc are still poorly understood. As a consequence, the current genotyping methods currently available are not sufficient to assess the genetic diversity of Bcc strains and predict the clinical outcome (Lee et al., 2017). By contrast, a genomic approach could provide more insights in Bcc evolution during chronic lung infections. A recent study describing the whole genome sequence analysis of 215 *B. cenocepacia* isolates, collected from 16 CF patients at different times of infection, demonstrated a considerable phenotypic and genotypic variability within single patients and confirmed that distinct lineages could follow distinct evolution patterns during chronic lung infection (Lee et al., 2017). Similar results were previously achieved by comparative genomic analysis of clinical isolates of *Pseudomonas aeruginosa* and *Burkholderia dolosa* from CF patients, thus confirming that these bacteria are indeed capable of accumulating different mutations at different stages of chronic infections (Lieberman et al., 2011; Markussen et al., 2014; Lee et al., 2017).

Genome-based taxonomic and phylogenetic analyses that have emerged as present identification standards can provide more accurate genotyping data of clinical isolates and also allow the identification of strains that have rapidly evolved after introduction of novel determinants by horizontal gene transfer (Juhas et al., 2009). For instance, a recent genome-based analysis of Bcc clinical isolates in India identified a previously unknown *B. cenocepacia* clone characterized by a novel genomic island (i.e., BcenGI15), very similar to that found in *Burkholderia pseudomallei* strain EY1, and so demonstrated transfer of genomic islands also between different pathogenic species within the *Burkholderia* genus (Patil et al., 2017).

## Resistance Mechanisms

Antibiotic resistance could be intrinsic or acquired. The first one is independent of antibiotic selective pressure and horizontal gene transfer, instead it is the result of inherent structural or functional characteristics. On the other hand, bacteria can acquire resistance to antibiotics, such as mutations in drug targets or transfer of resistance genes through phage-mediated transduction and mobile plasmids. Moreover, tolerance to antibiotics plays an important role in protecting bacteria during infections. This phenomenon is related to bacteria adaptation to environment, such as planktonic or sessile (biofilm) growth and presence of persister cells, and it can be due or not to mutations in target genes.

The four main mechanisms of antibiotic resistance are: prevention of access to target due to (1) reduced permeability of the cell envelope or to (2) increased efflux activity; (3) mutation in antibiotic target; (4) enzymatic modification or inactivation of the drug (hydrolysis or transfer of a chemical group). In addition, (5) the ability to form biofilms greatly enhance antibiotic resistance traits.

### Reduced Permeability of the Cell Envelope

In Gram negative bacteria the cell envelope is composed of an inner membrane, a periplasmic space and an outer membrane containing lipopolysaccharide (LPS) (Hamad et al., 2012). LPS comprises lipid A, core oligosaccharide (OS) and a polymer composed of glycan monomers called O-antigen (O-Ag). The lipid A-core OS and O-Ag are synthetized independently on the cytoplasmic side of the inner membrane, then they are joined in the periplasmic side and finally the complete LPS molecule is transferred to the outer membrane surface by a group of conserved proteins forming the lipopolysaccharide transport machinery (LPT) (Hamad et al., 2012). LPS undergoes further modifications, such as addition or removal of sugars, phosphates, or acyl groups allowing bacterial survival in stress conditions such as presence of antimicrobial peptides (Raetz et al., 2007). Among LPS alterations, the cationic substitution of phosphate groups by the addition of 4-amino-4-deoxy-L-arabinose (L-Ara4N), decreases the net negative charge of lipid A. *B. cenocepacia* is extremely resistant to antimicrobial peptides like polymyxin B. In *Burkholderia* spp. L-Ara4N is the main constituent of the lipid A and of the OS portion of the LPS (Olaitan et al., 2014). Deletion of the OS region leads to an increased binding of polymyxin B to *B. cenocepacia* cells and so increased sensitivity to polymyxin B (Ortega et al., 2009).

In addition, another important polymyxin B resistance determinant in *B. cenocepacia* is the alternative sigma factor RpoE involved in controlling the expression of a group of genes which are part of the extra-cytoplasmic stress response (Loutet and Valvano, 2011).

Finally, there are two other minor determinants of antimicrobial peptide resistance in *B. cenocepacia*. Two secreted zinc metalloproteases, ZmpA and ZmpB, are involved in the degradation of different antimicrobial peptides *in vitro*, however, *B. cenocepacia* knocked-out strains for one or both proteases did not show increased sensitivity. Loutet et al. (2011) demonstrated that antimicrobial peptide resistance could be found in a deep-rough LPS *B. cenocepacia* mutant in which the LPS molecule is truncated through the disruption of the ADP-L-glycero-D-manno-heptose synthesis.

### Overexpression of Efflux Pumps

In Gram negative bacteria efflux mechanisms play a major role in antibiotic resistance. Up to five families of transporters can be involved: the major facilitator superfamily (MFS), the ATP-binding cassette family (ABC), the small multidrug resistance family (SMR), the multidrug and toxic compound extrusion family (MATE), and the resistance nodulation division family (RND) (Li et al., 2015).

In *B. cenocepacia* 16 open reading frames encoding RND efflux pumps were identified. This type of transporters catalyzes substrate efflux via an H^+^ antiport mechanism. Genes are usually organized as operons and encode multimeric structures composed of an RND protein placed in the inner cellular membrane, an outer membrane protein (OMP) in the outer membrane and a membrane adaptor protein in the periplasmic region that connects the first two proteins (Li et al., 2015).

Different studies highlighted the key role of RND efflux pumps in drug resistance of *B. cenocepacia*, showing that in particular RND-3, RND-4, and RND-9 protect the bacterium from different compounds (Buroni et al., 2009, 2014; Bazzini et al., 2011). For example, *B. cenocepacia* cells treated with chlorhexidine overexpress several genes coding for drug resistance determinants such as RND efflux systems (Coenye et al., 2011). Analysis of RND mutants confirmed that some efflux transporters are involved in chlorhexidine efflux during planktonic growth (RND-1 and RND-4), while some others (RND-3 and RND-9) during sessile growth (Coenye et al., 2011).

Moreover, in order to better clarify the role of each RND efflux pumps in *B. cenocepacia*, MIC differences for certain antibiotics were evaluated using knock-out mutants for the 16 RND efflux systems, and analyzed during both planktonic and sessile growth. During planktonic growth RND-3 efflux pump was demonstrated to be crucial for resistance to ciprofloxacin and tobramycin, while RND-4 is important for extrusion of ciprofloxacin, tobramycin, minocycline, and chloramphenicol (Buroni et al., 2014). On the other hand, RND-8 and RND-9 play a key role in protection against tobramycin only during sessile growth (Buroni et al., 2014).

Mira et al. (2011) described the adaptive mechanisms that promote long-term colonization of *B. cenocepacia* in CF lungs by DNA microarrays transcriptomic analysis of two clonal variants isolated during long-term infection. Among the up-regulated genes in the most resistant strain, *mdtABC* and *bpeA*, were identified, respectively encoding RND-6 and RND-4 efflux pumps, *BCAM0201*, encoding an efflux system of the MFS; and *BCAM2188*, encoding a component of an ABC-transporter (Mira et al., 2011).

RND-4 and RND-9 were also involved in the resistance to two novel anti-*Burkholderia* experimental compounds (Scoffone et al., 2014, 2015).

A further study showed that in Bcc clinical isolates, RND-3 is the most up-regulated among the RND efflux systems due to mutations in its transcriptional regulator (Tseng et al., 2014).

Other efflux systems are also involved in *Burkholderia* antibiotic resistance: for example, the overexpression of *fsr* (fosmidomycin resistance gene) results in the upregulation of an efflux pump which leads to fosmidomycin resistance (Messiaen et al., 2011).

### Mutations in Antibiotic Cellular Target

Drug target modification is not the main resistance mechanism described in *B. cenocepacia*. A recent work analyzed the roles of the class 1 integron, the quinolone resistance-determining regions (QRDRs) of topoisomerases II and IV, in Bcc clinical isolates (Tseng et al., 2014). Levofloxacin resistance is due to accumulation of mutations in the QRDR genes encoding topoisomerases and efflux pump activation (Pope et al., 2008; Nikaido and Pages, 2012). Among 66 Bcc clinical isolates, 6 levofloxacin resistant strains were identified carrying single-base mutations in the QRDR region of the DNA gyrase subunit A (*gyrA* gene) at codon 81 (Gly81Asp), 83 (Thr83Ile), and 87 (Asp87His), respectively. No mutations were found in the QRDR region of the DNA topoisomerase IV subunit A (*parC* gene) (Tseng et al., 2014).

### Antibiotic Modification

This mechanism is commonly used by bacteria to achieve resistance to β-lactams and aminoglycosides.

β-lactams, such as penicillins, cephalosporins, clavams, carbapenems, and monobactams are inactivated by periplasmic β-lactamases through hydrolysis of the β-lactam ring (Bush and Fisher, 2011). Several β-lactamases were identified in *B. cenocepacia*: AmpC, hydrolysing expanded-spectrum cephalosporins; AmpD, a cell wall recycling enzyme (Holden et al., 2009); PenB, a Class A penicillinase extremely conserved across the Bcc (Poirel et al., 2009). β-lactams block the cell wall recycling system (Cho et al., 2014), but bacteria can detect them using PenR (AmpR), a transcriptional regulator that normally binds a precursor of peptidoglycan (i.e., UDP-MurNAc-pentapeptide) and represses the expression of *ampC*. PenR becomes a transcriptional activator after binding peptidoglycan degradation products like 1,6-anhydroMurNAc-peptides. Mutations in *ampD* have a key role in controlling expression of *ampC* and *penB* (Lee et al., 2015; Vadlamani et al., 2015). In a recent study, *ampD* mutations induced by ceftazidime were demonstrated to cause overexpression of PenB and AmpC β-lactamases encoding genes, hence causing a reduction to ceftazidime, cefotaxime, and meropenem susceptibility (Hwang and Kim, 2015).

A therapeutic strategy could be to block the activity of β-lactamases using β-lactamase inhibitors; a large number of class A enzymes are blocked by clavulanic acid, sulbactam or tazobactam, while class C (AmpC β-lactamases) and some class D enzymes are inhibited by avibactam (Drawz and Bonomo, 2010; Lagacé-Wiens et al., 2014). No inhibitors effective against class B β-lactamases have been described yet (Livermore et al., 2011; Aktaş et al., 2012). In this context, a recent work evaluated the effect of β-lactamase inhibitors on Bcc treated with β-lactam antibiotics (Everaert and Coenye, 2016). In *B. cenocepacia* LMG 16656 the authors did not observe any difference in the MIC for ceftazidime and cefepime when sulbactam, tazobactam, or avibactam were present. A possible explanation could be that in *B. cenocepacia* LMG 16656 the majority of the β-lactamases belong to the metallo-β-lactamase family (Winsor et al., 2008), so no effective inhibitors are available yet (Everaert and Coenye, 2016).

### Biofilm Formation and Persister Cells

When chronic infections are established in CF patients, bacterial cells are able to form biofilm, a matrix of extracellular polymeric molecules composed of DNA, polysaccharides, and proteins. In this context, they can further develop antibiotic resistance due to decreased antibiotic diffusion inside the biofilm matrix, reduction of nutrient availability resulting in metabolic changes that decrease antibiotic susceptibility, and, finally, phenotypic differentiation with appearance of persister cells which play key role in long-term infections (Mulcahy et al., 2010). Persister cells are a sub-population of cells that survive antibiotic treatment but, in contrast to resistant bacteria, this group does not express a specific resistance mechanism and their tolerance derives from physiological processes rather than genetic mutations (Allison et al., 2011). This kind of sub-populations is present in both sessile and planktonic cultures, but they are more difficult to eradicate after biofilm formation.

Persister cells are not mutated, but they are phenotypic variants of the wild type. After antibiotic treatment, these cells neither grow nor die and, and after drug removal, they start again to grow causing symptoms of infection. Van Acker et al. (2013) demonstrated that *B. cenocepacia* biofilms contain tolerant persister cells after tobramycin treatment.

Molecular mechanisms at the base of persistence are still largely unexplored, however, it was demonstrated that toxin–antitoxin modules (TA) play an important role not only in biofilm formation, gene regulation, programmed cell death and regulation of mobile genetic elements, but also in persistence mechanism (Gerdes and Maisonneuve, 2012). TA modules, abundant in bacteria and archaea, are two-component systems formed by a toxin that inhibits cell growth and by an antitoxin that controls toxin activity. There are five known types of TA loci. Toxin modules are always of protein nature in types I and III TA, while antitoxins are small RNAs that block toxin at translational and post-translational levels. Antitoxins of types II, IV, and V TA are proteins (Wang et al., 2012). Type II TA is well-characterized and usually the two genes are organized in an operon. During normal conditions, toxin and antitoxin form a complex that leads to inactivation of the toxin. Stress conditions induce degradation of the antitoxin and the toxin module regulates different cellular functions (Gerdes and Maisonneuve, 2012). Sixteen type II TA modules are present in *B. cenocepacia* J2315, 12 of which are conserved among *B. cenocepacia* strains. They were found to be up-regulated during biofilm growth (Van Acker et al., 2014). After treatment with tobramycin or ciprofloxacin, overexpression of toxins belonging to these TA systems contributes to persistence (Van Acker et al., 2014). TA modules could be an interesting target to treat chronic infections, but their redundancy is a challenging obstacle (Van Acker et al., 2014).

## New Therapies and Approaches to Overcome *B. cenocepacia* Infections

Until now, no standard treatment strategy has been described to eradicate *B. cenocepacia* chronic infections (Horsley and Jones, 2012; Regan and Bhatt, 2016). Several questions remain unanswered: the lack of correlation between *in vitro* and *in vivo* susceptibility data, the duration of therapy, the use of mono vs. combined antibiotic therapy (Gautam et al., 2015).

In general, the use of trimethoprim–sulfamethoxazole is recommended. If it cannot be administered, combinations containing first- and second-line agents, such as ceftazidime, meropenem, and penicillins (mainly piperacillin) can be considered according to the *in vitro* antimicrobial susceptibility patterns (Avgeri et al., 2009). As regarding penicillins (piperacillin–tazobactam and ticarcillin–clavulanate), different results were described by EUCAST and CLSI guidelines, so *in vitro* susceptibility assessment is required before administration.

Recommendations for infection prevention and control were reviewed by Saiman et al. (2014), while Tacconelli et al. (2014) described ESCMID guidelines for the management of infection control measures and reduce Gram negative transmission in hospitalized patients.

**FIGURE 1:**
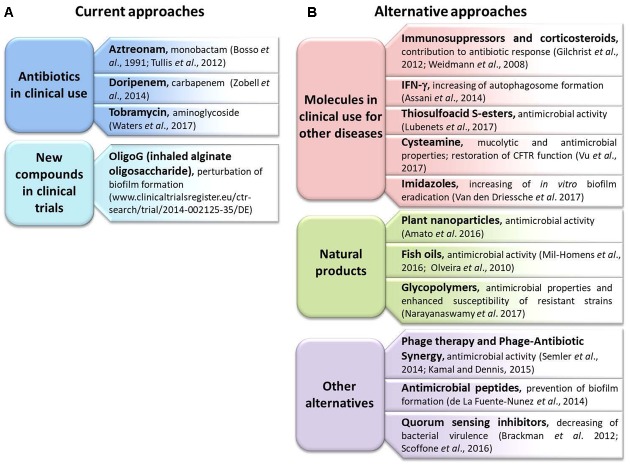
Summary of the new therapies and approaches described in the review. **(A)** The current approaches utilized in clinical practice; **(B)** new alternatives.

In this section we report a list of antibiotics in clinical use, a description of a new compound under clinical trial and several alternatives to standard antibiotics described in the literature during the last 5 years (**Figure 1**).

### Antibiotics in Clinical Use

#### Aztreonam

Aztreonam is a monobactam antibiotic approved in 1986 for the treatment of infections caused by Gram negative bacteria. It targets penicillin binding protein 3 (PBP3), thus inhibiting bacterial cell wall synthesis.

The solution for inhalation (AZLI; Cayston^®^; Gilead Sciences, Inc.) showed *in vitro* activity against *Burkholderia* spp. (Bosso et al., 1991) and a double-blind, placebo-controlled, 24-week trial of treatment was undertaken in CF patients with chronic Bcc infection (Tullis et al., 2012). Despite the fact that prolonged inhalation was well-tolerated, no significant improvement in the lung function was observed. The authors hypothesized that this could be due to non-study antibiotic use that may have confounded any potential therapeutic effect (Tullis et al., 2014).

#### Doripenem

Doripenem is a carbapenem approved in 2007 by the FDA. It is very similar to meropenem, but it shows a greater *in vitro* activity against *P. aeruginosa* (Hilas et al., 2008).

Intermittent extended infusion of doripenem was successfully used in a patient with history of methicillin-sensitive *S. aureus, S. maltophilia, Pseudomonas stutzeri*, and *B. cenocepacia* infections and showing meropenem shortage (Zobell et al., 2014). The report demonstrated that it is possible to administer doripenem also in children and that its intermittent extended infusion is effective to improve pulmonary function.

#### Tobramycin

Tobramycin is an aminoglycoside which prevents the formation of the 70S ribosomal complex. Tobramycin inhalation powder (TOBI Podhaler, Novartis) has been approved by FDA in 2011 to manage CF patients infected with *P. aeruginosa*.

Kennedy et al. (2015) showed the efficacy of high-dose tobramycin in reducing *Burkholderia* biofilm thickness *in vitro*, suggesting a possible role as a suppressive therapy in CF. Subsequently, a pilot, open-label clinical trial of TOBI Podhaler administered twice daily for 28 days in adults and children with CF and chronic *B. cepacia* complex infection was carried out (Waters et al., 2017). A decreased pulmonary bacterial burden and inflammation was observed, and the majority of patients showed no or mild adverse events. However, lung function was not significantly improved, possibly indicating that randomized controlled trials of longer duration are necessary (Waters et al., 2017).

### New Compounds in Clinical Trials

A search in the EU Clinical Trials Register^5^ and ClinicalTrials.gov^6^ using “cystic fibrosis and *Burkholderia*” keywords retrieved 12 and 8 results, respectively. Among the former, one was completed and the results are reported above in the “Antibiotics in clinical use” paragraph (Aztreonam), one had no results available and all the others were not pertaining to the search being not related to CF or to *Burkholderia* infections. Among the latter, one was about *Burkholderia dolosa* infections, two were completed and hence reported above in the “Antibiotics in clinical use” paragraph (Aztreonam and Tobramycin), for one the status had not been verified for more than 2 years, one was about *P. aeruginosa* infections, one was not related to infections but to microbiota of patients, and the last one had been withdrawn prior to enrollment.

For these reasons, the only trial worth reporting is the “Study of OligoG in cystic fibrosis subjects with *Burkholderia* spp. infection (SMR-2591)” which is ongoing in Germany. This is a randomized double-blind, placebo-controlled cross-over study of inhaled alginate oligosaccharide (OligoG) for the treatment of chronic *Burkholderia* infections in adults. Information about this trial is available at www.clinicaltrialsregister.eu/ctr-search/trial/2014-002125-35/DE. OligoG is a low-molecular-weight oligosaccharide enriched from sodium alginate polysaccharides. It is an oligomer composed of α-L-guluronic acid (>85%) and β-D-mannuronic acid (<15%). Alginate is produced by *P. aeruginosa* during its conversion to a mucoid phenotype in the formation of biofilms (Hentzer et al., 2001). Previous studies demonstrated an improved effect of antibiotics used in combination with OligoG due to perturbation of biofilm formation (Khan et al., 2012; Roberts et al., 2013). Patients (both males and females) with at least two sputum cultures positive for Bcc in the 12 months prior to the beginning of the study were recruited. Moreover, the eligibility criteria included the use of inhaled aztreonam. The main objective of this trial is to explore the efficacy of OligoG in reducing Bcc load in patients’ sputum. The study will also evaluate the effect of inhaled OligoG on lung function, quality of life, rheology, safety and patient compliance with treatment.

### Alternative Approaches

Molecules already in use to treat other clinical syndromes and products of natural origins are among the alternative approaches that can enhance current therapies or counteract the problem of resistance.

## Molecules in Clinical Use for Other Diseases

### Immunosuppressors and Corticosteroids

Immunomodulation has been suggested to contribute to the successful response to antibiotics in cases of cepacia syndrome (Gilchrist et al., 2012). This is probably due to the antagonist effect on the host response involved in the pathogenesis of cepacia syndrome. In one case, a patient was successfully treated with four intravenous antibiotics, oral corticosteroids, and cyclosporin (Gilchrist et al., 2012). In another case, four intravenous antibiotics, nebulised meropenem and tobramycin were used in combination with mycophenolate mofetil and tacrolimus (Weidmann et al., 2008). The role of immunosuppressant therapy in the management of *Burkholderia* infections is not completely understood, nevertheless it is worth considering this alternative approach for patients with poor prognosis.

### IFN-γ

Cystic fibrosis macrophages show a suboptimal IFN-γ response during *B. cenocepacia* infection causing deficient autophagosome formation, therefore it has been suggested that IFN-γ administration may help clearance of these bacteria (Assani et al., 2014). So far, this has been demonstrated only in cell culture models and the efficacy *in vivo* has yet to be determined.

### Thiosulfoacid *S*-Esters

Thiosulfoacid *S*-esters are organic compounds with established biological activity, already used as medicines, preservatives of vegetables, growth regulators, insecticides, and radioprotectors (Sotirova et al., 2012). Their chemical structure resembles that of natural compounds like garlic and onion derivatives (Block et al., 1996). In an attempt to find new molecules effective both against Gram-positive and negative microorganisms, Lubenets et al. (2017) synthesized *S*-esters of 4-acetyl, 4-trifluoroacetyl- and 4-(3-chloropropionylamino)-benzenethiosulfoacids and three of them showed MIC values within the micromolar concentration against *B. cepacia*. These molecules are examples of compounds worth further investigation to assess structure-activity relationships (SARs) and then prompt their practical application.

### Cysteamine

Cysteamine has been investigated as a potential enhancer of antimicrobial therapy in CF patients due to its ability to disrupt disulfide bonds, thus achieving a mucolytic activity and improving biofilm penetration (Gahl et al., 1985). This molecule is already approved for other diseases and its safety profile is known since 1994 (Gahl et al., 2007). Moreover, cysteamine showed antimicrobial activity against *P. aeruginosa*, so recently Fraser-Pitt et al. (2016) showed its effect in combination with antimicrobial agents used for the treatment of *Burkholderia*. In particular, they found that cysteamine was able to enhance the antimicrobial activity of tobramycin (even reversing resistance/insensitivity in 17 out of 36 strains), ciprofloxacin (reversing resistance/insensitivity in 10 strains), trimethoprim-sulfamethoxazole, but not ceftazidime. Furthermore, inhibition of bacterial biofilm was observed in presence of sub-inhibitory concentrations of cysteamine (Fraser-Pitt et al., 2016).

Interestingly, a very recent report showed that fatty acid cysteamine conjugates are able to promote transport of the misfolded CFTR to the surface of epithelial cells (Vu et al., 2017): in this way, besides having antimicrobial properties, cysteamine could be also useful to restore CFTR function in combination with potentiators and activators.

### Imidazoles

In an attempt to identify compounds able to increase the susceptibility of *B. cenocepacia* biofilms to tobramycin, Van den Driessche et al. (2017) screened a repurposing library containing non-toxic compounds with already known metabolic properties. A total of 60 compounds were identified. Among them, four antifungal imidazoles (namely econazole, miconazole, oxiconazole, and ketoconazole) were able to significantly decrease the concentration of tobramycin necessary to completely eradicate Bcc biofilms. However, no potentiating effect could be observed in a 3D long epithelial cells model, nor in *Galleria mellonella* and mouse models of infection (Van den Driessche et al., 2017).

## Natural Products

### Plant Nanoparticles

Essential oils (EOs) are complex extracts derived from aromatic plants comprising mixtures of aldehydes, terpenes, and phenols exhibiting broad spectrum antimicrobial activity (Bakkali et al., 2008). EO extracts containing carvacrol and thymol were shown to inhibit the growth of both clinical and environmental Bcc strains (Maida et al., 2014). Their mechanism of action relies on the partitioning of cytoplasmic membranes resulting in increased permeability, depletion of proton gradients, and disruption of ATP synthesis, ultimately leading to the death of bacterial cells.

First evidencies of antibacterial efficacy of thymol/carvacrol-loaded polymer nanoparticles against *B. cenocepacia* were reported by Amato et al. (2016) using polymer nanoparticles developed to overcome EO hydrophobicity, volatility and instability.

### Fish Oils

Polyunsaturated fatty acids (PUFAs), such as essential omega-3 and omega-6 fatty acids, were shown to have antimicrobial activity, probably by disturbing cell membrane structures and associated functions like electron transport, proton gradient and enzymatic activities (Desbois and Smith, 2010). In particular, fish oils are a source of the omega-3 PUFA eicosapentaenoic (EPA) and docosahexaenoic acid (DHA). Different papers described the use of omega-3 PUFAs to control *P. aeruginosa* infections *in vivo* (Tiesset et al., 2011; Caron et al., 2015) and *in vitro* (Tiesset et al., 2009), as well as *B. cenocepacia* infections (Mil-Homens et al., 2012). In particular, Mil-Homens et al. (2016) reported on their efficacy in treating *Burkholderia* infections and also as prophylactic therapy, using *G. mellonella* as infection model. Interestingly, as CF patients are deficient in fatty acids metabolism, administration of omega-3 PUFAs could be beneficial both to aid infection treatment and to improve respiratory, inflammatory, and nutritional parameters (Olveira et al., 2010).

### Glycopolymers

Poly (acetyl, arginyl) glucosamine (PAAG) is a polycationic polysaccharide which represents a novel class of glycopolymers with antibacterial properties as well as synergy with antibiotics *in vitro* (Siedenbiedel and Tiller, 2012). Its mechanism of action relies on the interaction with the outer membrane of Gram negatives causing a depolarization which results in leakage of the intracellular contents and death. In particular, divalent cations located within the bacterial outer membrane electrostatically bind the LPS by the anionic phosphate groups (Rajyaguru and Muszynski, 1997). Recently, Narayanaswamy et al. (2017) showed that PAAG is effective to treat lung infections caused by Bcc in CF patients in combinations with Meropenem and Tobramycin as it enhances susceptibility of resistant strains.

## Other Alternatives

### Phage Therapy and Phage-Antibiotic Synergy

Phage therapy is the therapeutic application of bacterial viruses (bacteriophages) commercially developed in the 1930s to reduce or eliminate infection (Gravitz, 2012). This is considered a valuable alternative to chemotherapeutic agents due to the specificity of phages toward bacterial cells and their exponential replication highly enhancing the therapeutic potential (Alisky et al., 1998).

Semler et al. (2014) compared the activity of phages delivered as aerosol to that of phages delivered via an intraperitoneal (i.p.) route to treat *B. cenocepacia* infections in a murine model. Their results showed that infected mice receiving aerosolized phage treatments exhibited a significant decrease in bacterial loads within the lungs, which could not be observed in those receiving treatment via i.p. injection. In this way, they demonstrated that aerosolization provides more widespread and uniform particle deposition, while i.p. delivered phages reach only certain areas in the lungs and may be unable to co-localize with the bacteria in the lung lumen (Semler et al., 2014).

Phage-antibiotic synergy (PAS) is the effect that some antibiotics exert on the ability of phages to form larger plaque under sub-lethal concentrations of the compound itself (Comeau et al., 2007). Ciprofloxacin, meropenem, and tetracycline exhibited PAS in combination with two *B. cenocepacia* phages enlarging plaque size (Kamal and Dennis, 2015). In particular, presence of ciprofloxacin and meropenem leads to the formation of elongated or filamented cells, and so phages may have increased access to phage receptors; moreover, cell clustering in presence of tetracycline allows phages to travel laterally across adjoined cell surfaces, again enhancing contact with phage receptors. PAS effect was not altered when treating antibiotic resistant cells, thus encouraging the use of this alternative method also with drug resistant strains (Kamal and Dennis, 2015).

### Antimicrobial Peptides

Cationic antimicrobial peptides (CAMPs) have been isolated from very different organisms such as microorganisms, invertebrates, plants, and mammals. They are able to establish strong non-specific, hydrophobic and electrostatic interactions with bacterial cytoplasmic membranes (Godballe et al., 2011) and prevent cell adhesion via electrostatic bonds (Overhage et al., 2008).

Antimicrobial peptides able to block *B. cenocepacia* biofilm formation were described in de la Fuente-Núñez et al. (2014). These peptides are similar to CAMPs but have different SAR. They exert their activity by blocking the stringent response mediated through (p)ppGpp, two small signaling nucleotides (guanosine 5′-diphosphate 3′-diphosphate or ppGpp and guanosine 5′-triphosphate 3′-diphosphate or pppGpp) (Potrykus and Cashel, 2008). These antimicrobial peptides showed activity against both Gram negative and positives bacteria. The peptide described by de la Fuente-Núñez et al. (2014) directly interacts with (p)ppGpp and is able to cross bacterial membranes to reach the cytoplasm. It can prevent biofilm formation and promote biofilm dispersal, and also cell death in biofilms at concentrations sub-lethal for planktonic cells (de la Fuente-Núñez et al., 2014).

### Quorum Sensing Inhibitors

In order to attenuate bacterial virulence, several quorum sensing inhibitors (QSI) have been developed in the last 15 years. Among them, analogs of the signal molecule Acyl-homoserine lactone (AHL) are obtained by modification of the acyl side chain, the lactone moiety (Ni et al., 2009) or the central amide moiety (Boukraa et al., 2011). Valuable AHL analogs, able to affect *B. cenocepacia* (as well as *P. aeruginosa*) QS and, in turn, inhibit and eradicate biofilm, have been described by Brackman et al. (2012).

More recently, another class of QSIs, namely diketopiperazines, was shown to inhibit the activity of the AHL synthase CepI of *B. cenocepacia* (Scoffone et al., 2016). These compounds interfered with the production of virulence factors, such as proteases and siderophores, as well as with biofilm formation, and showed good *in vivo* activity using a *Caenorhabditis elegans* infection model. Study results suggested that they could be considered for *in vivo* treatments combined with established or novel antimicrobials (Scoffone et al., 2016).

## Conclusion

Even if the epidemiology of CF pathogens is continuously changing and *B. cenocepacia* is not one of the prevalent bacteria colonizing the lung of patients, it still remains a major threat due to its extreme resistance to antibiotics and its ability to cause a life-threatening CF complication known as cepacia syndrome. In the last years, several studies were aimed at deciphering the principal mechanisms of resistance in *Burkholderia*, however, many aspects have not yet been fully understood. This has important repercussions on the choice of antibacterial drugs to be used and on our knowledge of the physiology of a bacterium with a wide genome yet to be fully explored.

The lack of a standard therapy regimen makes the treatment of Bcc infections challenging and more studies are needed to improve survival and quality of life of CF patients. On the contrary, evidence-based eradication guidelines are available for *P. aeruginosa* allowing standardization of antimicrobial treatment and hence improving management of these infections (Mogayzel et al., 2014). The introduction of correctors and potentiators of the CFTR defects requires more studies of drug-drug interactions to predict treatment efficacy, however, this is not possible while a standard therapy for Bcc is not available. Since few patients are infected by *B. cenocepacia*, an increased effort to coordinate clinical trials and observational studies among different hospitals is needed, maximizing data necessary to standardize methods for evaluation of antibiotic susceptibility.

Since the development of new drugs is not trivial, it is necessary to improve the use of existing therapies while coordinating trials of new molecules which showed their potential *in vitro* and in animal models.

## Author Contributions

All authors listed have made a substantial, direct and intellectual contribution to the work, and approved it for publication.

## Conflict of Interest Statement

The authors declare that the research was conducted in the absence of any commercial or financial relationships that could be construed as a potential conflict of interest.

## Footnotes

1 http://www.cysticfibrosis.ca/uploads/Registry%20Report%202015/2015%20Registry%20Annual%20Report%20EN.pdf

2 https://www.cff.org/Our-Research/CF-Patient-Registry/2015-Patient-Registry-Annual-Data-Report.pdf

3 https://www.ecfs.eu/sites/default/files/general-content-files/working-groups/ecfs-patient-registry/ECFSPR_Annual%20Repor

4 http://pubmlst.org/bcc/

5 https://www.clinicaltrialsregister.eu/ctr-search/search

6 https://clinicaltrials.gov/ct2/home
